# Supplementary material for: Characterization of ori and parS-like functions in secondary genome replicons in Deinococcus radiodurans
Source: Life Sci Alliance. 2020 Nov 16;4(1):e202000856. doi: 10.26508/lsa.202000856 (PMC7671480; doi:10.26508/lsa.202000856)
Supplement: Supplementary file 1 [file LSA-2020-00856_TableS1.doc]

**Table S1 :** List of oligonucleotides used in this study

| Primer Name | Oligonucleotide Sequences | Purpose/Plasmid |
| --- | --- | --- |
| cisIIFw | 5' GCTCTAGAGATCAAATCTTTTAAAG3' | pNOKcisII, EMSA (cisII; full length) |
| cisIIRw | 5' GCTCTAGACTTAATAGACCTGTAATTG 3' |
| cisMPFw | 5’ GCGGGCCC CAAGGACGGCTTCTCTC 3’ | pNOKcisMP, EMSA (cisMP; full length) |
| cisMPRw | 5' CG GAATTC AGTTGCAGACCATAGGGGT 3' |
| CIIintFw1 | 5’ TCCCCGCGGTCCAAATG 3’ | EMSA (cisII; 8 repeats) |
| CIIintFw2 | 5’TCCCCGCGGCAAACGGCCCA 3’ | EMSA (cisII; 3 repeats) |
| CIIintFw3 | 5’CTCCACAAAGTGCCACAGGTAATTCCACAAAGTGCCACAGGC 3’ | EMSA (cisII; 2 repeats) |
| CIIintRw3 | 5’GCCTGTGGCACTTTGTGGAATTACCTGTGGCACTTTGTGGAG 3’ |
| CIIintFw4 | 5’ CTCCACAAAGTGCCACAGGTG 3’ | EMSA (cisII; 1 repeat) |
| CIIintRw4 | 5’ CACCTGTGGCACTTTGTGGAG 3’ |
| CMintFw1 | 5’ GCGGGCCCTTTTGCACGTTG 3’ | EMSA (cisMP; 5 repeats) |
| CMintFw2 | 5’ GCGGGCCCGTCTACAAAGAG 3’ | EMSA (cisMP; 3 repeats) |
| CMintFw3 | 5’ACGCAAAGGTGTCGCTATTTTGACCCCAAATCCCGCAAAGGTGTCGCTAT 3’ | EMSA (cisMP; 2 repeats) |
| CMintRw3 | 5’ATAGCGACACCTTTGCGGGATTTGGGGTCAAAATAGCGACACCTTTGCGT 3’ |
| CMintFw4 | 5’ CCCGCAAAGGTGTCGCTAGG 3’ | EMSA (cisMP; 1 repeat) |
| CMintRw4 | 5’ CCTAGCGACACCTTTGCGGG 3’ |
| CIIUPFw. | 5’ GGGGTACC TCGGTCACGTCGTATGC 3’ | pNOKCII |
| CIIUPRw | 5’ CGGAATTC CCTATGATGATGATCATC 3’ |
| CIIDNFw | 5’ CGGGATCC TTTGTGCTGAAGAATCATC 3’ |
| CIIDNRw | 5’ GCTCTAGA AAGGCTAGGCGGACTATC 3’ |
| CMPUPFw | 5’ GGGGTACCGACAGAAGTCTTACGGCC 3’ | pNOKCMP |
| CMPUPRw | 5’ CGGAATTCAGCGACACCTTTGCGGGA 3’ |
| CMPDNFw | 5’ CGGGATCCCTTGTAAAATTCACCAAC 3’ |
| CMPDNRw | 5’ GCTCTAGAGCCCGAGAGAAGGGGGAC 3’ |
| pETDAF | 5’ CG GGATCCGTGCGCAAAAACGTCTC 3’ | pETDnaA |
| pETDAR | 5’ GC GAATTCTTACGCCCCGACTTCTTC 3’ |
| RTZ Fw | 5' ATCAAGGAATATCTCGA 3' | Quantitative PCR study |
| RTZ Rw | 5' CAGCTTTTCGTTGTTCACC 3' |
| RTEFw | 5’ TTGAGCGTCCCCGCGCC3’ |
| RTERw | 5’GTGCCCGACGAGGTAGA3’ |
| RTKFw | 5’GTGCGGCGCCTGCAACGC3’ |
| RTKRw | 5’CCGCCGATAGACAGAATC3’ |
| A155RTFw | 5’ TTGGCGCATTTTCCCGGC 3’ |
| A155RTRw | 5’ CAGCAGGTTGATCGCCTG 3’ |
| PprARTFw | 5’ GTGCTACCCCTGGCCTT 3’ |
| PPRARTRW | 5’ GCGGCCATCGGTCAGAAT 3’ |
| A182RTFW | 5’ ATTCTGGGGCCGGAGCTG3’ |
| A182RTRW | 5’CTTGCGTTCCCCCGGCGG 3’ |
| B03RTFw | 5’ CTGAGTCCTGACGAGTCC 3’ |
| B03RTRw | 5’ TTCCGGGTGACGCAGCAG 3’ |
| B30RTFw | 5’ ATGAGCCGCAAGTTGCCG3’ |
| B30RTRw | 5’ GCCAGCGAGGCGGCTCGC 3’ |
| B104RTFw | 5’ ATGAAAACTCTTGAGGC3’ |
| B104RTRw | `5 GCCGAGGAAAACGTCCAG 3’ |
| C01RTFw | 5’ ATGTGCTCGCCTCCTAGA 3’ |
| C01RTRw | 5’ TCACTGTGAAACCTGATC 3’ |
| C18RTFw | 5’ ATGACACAGACGCGGCG 3’ |
| C18RTRw | 5’ GTCCGCGAGGCGCATCAT 3’ |
| C34RTFw | 5’TGGTGGCATTTCTCCGTG3’ |
| C34RTRw | 5’ CACTGAAATACCCCAGCC3’ |
| nptFw | 5’GCACGGTGGCCGAGTGG3’ |
| nptmidRw | 5’AACATCATTGGCAACGCT3’ |
| blaRTFw | 5’GGATCATGTAACTCGCCT3’ |
| blaRTRw | 5’TTACCAATGCTTAATCAGTGAGG3’ |
| ChI(1.5 ̊ )Fw | 5’GCTCTAGAGTCGACGCCTCTTTTCACCGCAAAG 3’ | p44Ch1 |
| ChI(1.5 ̊ )Rw | 5’AAAAGTACTCATATGCCGGACATGTCCGGGCGC3’ |
| Ch2(4 ̊ )Fw | 5’GCTCTAGAGTCGACGGCAGCGAGGTCAGGAAG 3’ | p44Ch2 |
| Ch2(4 ̊ )Rw | 5’AAAAGTACTAAGCTTACGTCCGGCAAGCACCTG3’ |
| MP(4.4 ̊ )Fw | 5’GCTCTAGAGTCGACGAAGCTGGTAAAACTTTG3’ | p44MP |
| MP(4.4 ̊ )Rw | 5’AAAGTACTCATATGTACGCCCGAAAGCCTACAG3’ |
| SpecFw | 5’ATGAGGGAAGCGGTGATC3’ | p44SCh1, p44SCh2 and p44SMP construction and Diagnostic PCR |
| SpecRw | 5’TTATTTGCCGACTACCT3’ |
| TetRscIApIFw | 5’CGGAGCTCGGGCCCGTGAGATTAGATAAAAG3’ | pDTRGFP & pRADTRGFP |
| TetRSalIRw | 5’GCGTCGACAGACCCACTTTCACATTTAAG3’ |
| GFPXbaIRw | 5’GCTCTAGATTATTTGTATAGTTCATCCA3’ |
| AmpRw | 5’TTACCAATGCTTAATCAGTGAGG3’ | Diagnostic PCR |
| Dr0010F | 5’GTGAAATCACCGCTTCCAATG3’ |
| DrA005F | 5’ATGAAAGCAATTGTCTGGCAAG3’ |
| DrB003F | 5’TTGGCCCTGCAACCGGAAC3’ |
| A2AfIIIFw | 5’CCCACGTGTTCTGCGCCGCGCTTTAGC 3’ | Primers for non-specific DNA in EMSA with drDnaA/ParBs |
| A2SxIRw | 5’GTACGACCTGGTCACGTCGGCATGAACGGGT 3’ |
| 260bpFw | 5'ATCAAGGAATATCTCGA3' (RTZFW) |
| 260bpRw | 5'CAGCTTTTCGTTGTTCACC3' (RTZRW) |
| 100bpFw | 5'ATCAAGGAATATCTCGA3' (RTZFW) |
| 100bpRw | 5’GGGCAATTTCGGCCACGAC3’ |
