# Supplementary material for: Characterization of ori and parS-like functions in secondary genome replicons in Deinococcus radiodurans
Source: Life Sci Alliance. 2020 Nov 16;4(1):e202000856. doi: 10.26508/lsa.202000856 (PMC7671480; doi:10.26508/lsa.202000856)
Supplement: Supplementary file 2 [file LSA-2020-00856_TableS2.doc]

**Table S2: List of bacterial strains and plasmids used in this study**

| **Bacterial strains** | | | **Genotype** | **Source** | |
| --- | --- | --- | --- | --- | --- |
| *D. radiodurans* R1 | | | Wild type strain ATCC13939 | Lab stock | |
| *ΔcisII* | | | *cisII* (region; 150-515) replaced with *nptII* cassetess from chromosome II of *D. radiodurans* R1 (Kan^R^) | This study | |
| *ΔcisMP* | | | *cisMP* (region; 177403- 400) replaced with *nptII* cassetess from megaplasmid of *D. radiodurans* R1 ( Kan^R^) | This study | |
| R1::ChrI-*tetO* | | | p44SCh1 plasmid integrated at 1.5˚ position in chromosome I of wild type (Spec^R^) | This study | |
| Δ*cisII*::ChrI-*tetO* | | | p44SCh1 plasmid integrated at 1.5˚ position in chromosome I of  *ΔcisII* (Kan^R^; Spec^R^) | This study | |
| Δ*cisMP*::ChrI-*tetO* | | | p44SCh1 plasmid integrated at 1.5˚ position in chromosome I of  *ΔcisMP* (Kan^R^; Spec^R^) | This study | |
| R1::ChrII-*tetO* | | | p44SCh2 plasmid integrated at 4˚ position in chromosome II of wild type (Spec^R^) | This study | |
| Δ*cisII*::ChrII-*tetO* | | | p44SCh2 plasmid integrated at 4˚ position in chromosome II of  *ΔcisII* (Kan^R^; Spec^R^) | This study | |
| Δ*cisMP*::ChrII-*tetO* | | | p44SCh2 plasmid integrated at 4˚ position in chromosome II of  *ΔcisMP* (Kan^R^; Spec^R^) | This study | |
| R1::MP-*tetO* | | | p44SMP plasmid integrated at 4.4˚ position in megaplasmid of wild type (Spec^R^) | This study | |
| Δ*cisII*::MP-*tetO* | | | p44SMP plasmid integrated at 4.4˚ position in megaplasmid of  *ΔcisII* (Kan^R^; Spec^R^) | This study | |
| Δ*cisMP*::MP-*tetO* | | | p44SMP plasmid integrated at 4.4˚ position in megaplasmid of  *ΔcisMP* (Kan^R^; Spec^R^) | This study | |
| *ΔrecA* | | | Deinococcal *recA* gene disrupted with chloramphenicol resistance gene cassettes (Cam^R^) | Lab stock | |
| *E. coli* NovaBlue | | | *end*A1 *hsd*R17*(r _K12_ − m _K12_ ^+^) sup*E44 *thi-1 rec*A1 *gyr*A96 *rel*A1 *lac*F’'*[pro*A^+^B*^+^ lac*I^q^ *Z∆*M15*::*T*n*10 ] (Tet^R^) | NEB Inc., | |
| *E. coli* BL21(DE3) | | | *fhu*A2 *(lon)omp*T *gal*(λDE3)*(dcm) ∆hs*dS | Lab stock | |
| **Plasmids** | | | | | |
| **Sr No.** | **Plasmids** | **Characteristics** | | **Sources** | **MW of**  **Protein**  **(~kDa)** |
| 1 | pET28a(+) | ~ 5.3 kb plasmid; N-terminal 6XHis tag (Kan^R^) | | Novagen | - |
| 2 | pETDnaA | pET28a(+) carrying Dr_0002 at *Bam*HI and *Eco*RI | | This study | ~ 53 kDa |
|  | pETB2 | pET28a(+) carrying Dr_A0002 at *Nde*I and *Xho*I | | Maurya et al., 2019a | ~33 kDa |
|  | pETB3 | pET28a(+) carrying Dr_B0002 at *Nde*I and *Xho*I | | Maurya et al., 2019a | ~32 kDa |
| 3 | pNOKOUT | A deinoccocal suicidal vector; Kan^R^ | | Kahirnar et al., 2008 | - |
| 4 | pNOKcisII, | pNOKOUT carrying full length *cisII* at *Xba*I site | | This study | - |
| 5 | pNOKcisMP | pNOKOUT carrying full length *cisMP* at *Apa*I-*Eco*RI sites | | This study | - |
| 6 | pNOKCII | pNOKOUT carrying ~500 bps upstream at *Kpn*I & *Eco*RI and ~500 bps downstream at *Bam*HI & *Xba*I of *cisII* element (Kan^R^) | | This study | - |
| 7 | pNOKCMP | pNOKOUT carrying ~500 bps upstream at *Kpn*I & *Eco*RI and ~500 bps downstream at *Bam*HI & *Xba*I of *cisMP* element (Kan^R^) | | This study | - |
|  | pLAU44 | Plasmid with an array of 240 repeats of *tetO* (Amp^R^& Gen^R^) | | Lau *et al*., 2003 | - |
|  | p44SCh1 | pLAU44 with region (10713-11715) from chromosome I at *Xba*I-*Sca*I, with Spectinomycin cassette from pVHS559 at *Nhe*I-*Xho*I | | This study | - |
|  | p44SCh2 | pLAU44 with region (4695-c5691) from chromosome II at *Xba*I-*Sca*I, with Spectinomycin cassette from pVHS559 at *Nhe*I-*Xho*I | | This study | - |
|  | P44SMP | pLAU44 with region (2203-3000) from megaplasmid at *Xba*I-*Sca*I, with Spectinomycin cassette from pVHS559 at *Nhe*I-*Xho*I | | This study | - |
|  | pDSW208 | PDSW208-MCS-*gfp* (fusion vector) (Amp^R^) | | Weiss et al., 1999 | ~27 kDa |
|  | pLAU53 | pBAD24 backbone containing *lacI*-eCFP and *tetR*-eYFP behind the same *araB* promoter, (Kan^R^ & Amp^R^) | | Lau et al., 2003 | - |
|  | pDTRGFP | pDSW208 with *tetR* from pLAU53at *Sac*I-*Sal*I to give *gfp-tetR* | | This study | ~48 kDa |
|  | pRADTRGFP | pRADgro with *gfp-tetR* at *Apa*I-*Xba*I from pDTRGFP | | This study | ~48 kDa |
